# Features and Limitations of LitCovid Hub for Quick Access to Literature About COVID-19

**DOI:** 10.4274/balkanmedj.galenos.2020.2020.4.67

**Published:** 2020-06-01

**Authors:** Morteza Arab-Zozani, Soheil Hassanipour

**Affiliations:** 1 Social Determinants of Health Research Center, Birjand University of Medical Sciences, Birjand, Iran; 2 Gastrointestinal and Liver Diseases Research Center, Guilan University of Medical Sciences, Rasht, Iran

To the Editor,

In mid-December 2019, an outbreak of severe acute respiratory syndrome coronavirus-2 (SARS-CoV-2), which began in Wuhan, China, has spread throughout the country and COVID-19 was announced as a pandemic disease (1). Obtaining the right evidence in the shortest possible time has always been a concern for researchers, policymakers, and decision-makers, especially in times of crisis (2). From the very first days, researchers started to publish articles about COVID-19, and the number of articles increased daily. Also, various publications have devoted sections to the disease and tried to provide up-to-date information. Since searching databases require special skills, accessing the articles related to this diseases is not easy, and initiatives in this area can be essential in terms of obtaining accurate and timely information (3).

One of the most interesting initiatives, which started in the early days, was the launch of a dedicated LitCovid hub in PubMed (available at https://www.ncbi.nlm.nih.gov/research/coronavirus/) to track and gather up-to-date information about 2019 novel coronavirus. The hub has unique features and covers almost a high percentage of articles published about COVID-19 (4). This hub is updated daily with newly published articles and includes the most comprehensive collection of international research papers so far on the new coronavirus disease, COVID-19. According to the authors, this hub has a more sophisticated search function than available resources and identifies about 35% more relevant articles compared to formal keyword-based searches for entries such as COVID-19, nCOV, 2019-nCoV, and other related search terms. Also, the articles available on this hub are categorized by several topics, including general information (general information and news), mechanism (symptoms, clinical characteristics, and findings from sequence and image analysis), transmission (characteristics and modes of COVID-19 transmission, such as human-to-human), treatment (treatment strategies, therapeutic procedures, and vaccine development), case reports (descriptions of specific patient cases), and epidemic forecasting (modeling and estimating the trend of COVID-19 spread) (4).

We analyzed the publication activity concerned with COVID-19 in LitCovid hub from January 17 to April 05, 2020. Based on a search conducted on April 06, 2020, the total number of articles on this hub was 3011, which was much more than what databases such as Scopus and Web of Sciences (WoS) had in the same period. During this period, 1639 articles were searched in Scopus and 522 articles, in WoS, using conventional keywords. This suggests that more specific articles can be retrieved in this hub, according to the developers.

Based on our search, most articles have been published on April 03, 2020 (9.33%) (Figure 1). Most of the published articles were related to China (30.68%), followed by the United States (3.15%) and Italy (3.12%). Most of the articles were published in BMJ (5.87%), Journal of Medical Virology (3.48%), and Lancet (3.12%). The largest number of published articles (21.32%) was in the treatment category (Table 1).

An important feature of this hub is that it is proprietary and does not require an initial search to retrieve articles about COVID-19. Another important feature is that it is open source, in addition to being updated daily. This hub is also notable for features such as importing records in two ways, RIS and TSV formats, categorizing articles according to subject areas, and having a link for text and data mining, which researchers find useful (4). Of course, publishers like Nature have also categorized and published their publishers’ articles, but the comprehensiveness of this hub is much greater (5).

Indeed, there are some limitations. For example, this hub is based on PubMed database, and articles in other journals that are not indexed in this database may not be traceable. Another limitation is the lack of a dedicated search feature for the content of this hub. It is suggested that these limitations be addressed in the near future and that more specific areas be added to this hub depending on the type of articles, because, over time, rapid access to studies such as clinical trials will be of utmost importance.

## Figures and Tables

**Table 1:**
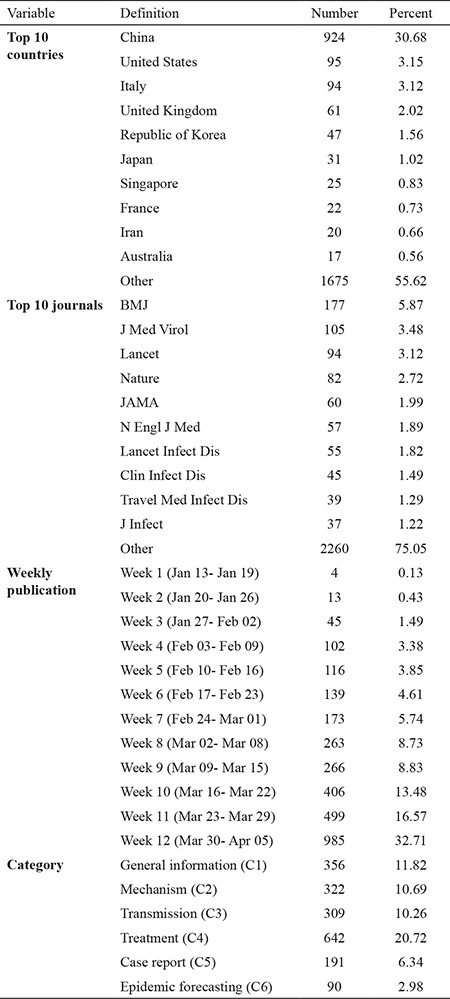
Summary of the characteristics of included studies

**Figure 1:**
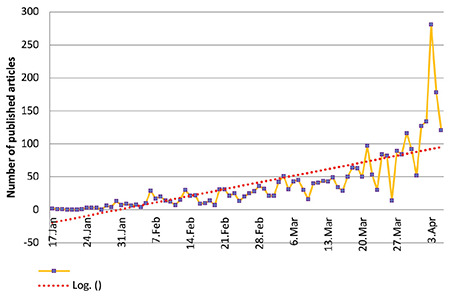
Distribution of published articles by day.
